# Vav Proteins' Role in Skin Cancer

**DOI:** 10.1371/journal.pbio.1001617

**Published:** 2013-07-23

**Authors:** Robin Mejia

**Affiliations:** Freelance Science Writer, Albany, California, United States of America

The most common form of skin cancer, squamous cell carcinoma usually isn't deadly. However, neither is it benign; in some cases it can be aggressive and spread to other organs. And much is still not known about the signaling pathways that enable the formation of this kind of cancer.

**Figure:**
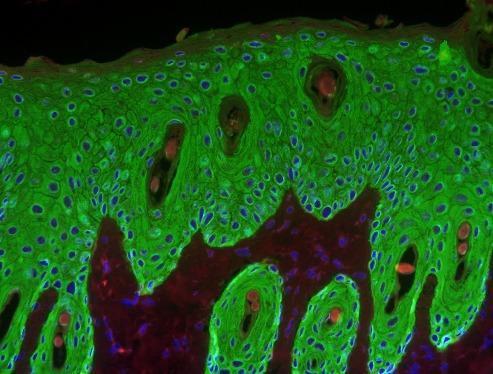
Skin section showing the proliferative response induced by a tumor promoter in the epidermis of a wild-type mouse. **Keratinocytes are shown in green. As reported in this issue, this reaction is severely reduced in Vav-deficient mice.** Image generated by M. Menacho-Márquez.

Rho GTPases, which influence gene expression, cell proliferation, and programmed cell death, show high levels of activity in many cancers, including skin cancer. Researchers believe a family of compounds called GDP/GDT exchange factors (GEFs) are critical to Rho GTPase activity in tumor development and maintenance; however, because there are so many GEFs, determining which ones are essential and what their exact roles are have been open questions. In this issue of *PLOS Biology*, Mauricio Menacho-Márquez and colleagues start to provide answers. The researchers describe studies showing that GEFs of the Vav subfamily play important roles in the development of skin cancer tumors in mice.

To do this, the scientists worked with knockout mice deficient for Vav2 and Vav3 and also with tissue cultures that were deficient for the two proteins. First, using wild-type and knockout mice, the researchers used two methods involving powerful carcinogens to promote the development of squamous cell carcinomas. In one case, mice were subjected to repeat applications of 7,12-dimethylylben[*a*]antracene (DMBA). The knockout mice had a 2-fold reduction in total tumors, indicating that the knocked out genes (Vav2 and Vav3), were important to tumor formation. When the mice were treated with DMBA followed by 12-O-tetradecanoylphorbol-13-acetate (TPA), the difference was even starker. The knockout mice had a 5-fold lower rate of tumors. The knockout mice showed normal skin development, suggesting that Vav2 and Vav3 were important for cancer formation but not necessary for normal skin development.

Additional experiments sought to explain how the Vav proteins promote tumors. Looking at the immediate reactions to DMBA, the scientists found that the compounds caused a 2-fold increase in programmed cell death in the knockout mice. In tissue culture, deficient keratinocytes, the main cell type of the epidermis, also suffered higher rates of apoptosis. This fits with the lower rates of tumor development: if the cells die, they can't become cancerous. The team also showed that TPA has a different effect on wild-type and knockout mice; knockouts do not have the normal proliferative and inflammatory responses to the chemical, indicating that Vav2 and Vav3 are needed for these responses and subsequent tumor formation. Importantly, this is seen even in mice with a normal blood formation system, indicating that it's the role of Vav2 and Vav3 in the epidermis that matters.

Further experiments demonstrated that Vav proteins influence intrinsic signaling programs in skin cells. Specifically, they showed that when TPA was added to keratinocyte cell cultures, there was reduced activation of proteins involved in cell cycle progression such as extracellular-regulated kinase (Erk), a serine/threonine kinase, and the signal transducer and activator of transcription (Stat3), a transcription factor. The phosphorylation levels of Erk and Stat3 could be rescued by the re-expression of Vav2 or Vav3 in these cells, indicating that those defects were a direct consequence of the absence of these proteins in skin cells.

Microarray experiments revealed that Vav proteins are involved in the activation of a large biological program composed of extracellular factors that influence the behavior of epithelial cells and their neighbors, such as inflammatory and other tumor-associated stromal cells. Co-culturing of Vav-deficient keratinocytes in the presence of wild-type cells eliminated both their survival and proliferative defects, indicating that Vav proteins play a critical role. This extracellular signaling program was not conserved in the Vav2/Vav3-dependent transcriptome that the same group has described recently in mouse breast cancer cells, supporting the idea that this biological program is specific to the epidermal cells. By contrast, the Vav2/Vav3-dependent gene signature was found conserved in fully developed tumors, indicating that it may also play roles in tumor maintenance and/or progression.

Taken as a whole, these experiments indicate that Vav2 and Vav3 play important roles in the initiation and development of skin cancers, and that they also promote longer-term changes in cellular signaling that support cellular survival of DNA damage, with increases in proliferation and the development of an inflammatory environment that can lead to tumor formation.

The researchers caution that these experiments have likely identified just “the tip of the iceberg of this biological program,” noting that the Vav2- and Vav3-dependent transcriptome they identified encodes many potentially pro-cancerous factors that have not yet been identified. Future experiments may demonstrate even more widespread reprogramming of tissue microenvironment. However, the results presented here suggest that Vav proteins may provide useful pharmacological targets. Once again, more research is needed: these studies suggest preventive therapies; further studies will show if the Vav systems would make useful targets for cancer treatments.

**Menacho-Márquez M, García-Escudero R, Ojeda V, Abad A, Delgado P, et al. The Rho Exchange Factors Vav2 and Vav3 Favor Skin Tumor Initiation and Promotion by Engaging Extracellular Signaling Loops. doi:10.1371/journal.pbio.1001615**

